# Recruiting by the New Method

**Published:** 1917-08-04

**Authors:** 


### RECRUITING BY THE NEW METHOD.

### Civilian Doctors to Replace Military Officers.

Expedition is not a note which is commonly placed to the credit of official inquiries. On the contrary, cynical comment is disposed to say not only that such inquiries show a large capacity for delay but also that it is this quality which endears them to the official mind. No charge of this order will lie against the Parliamentary Committee recently appointed to investigate the conditions of present-day recruiting for the Army. The Committee has indeed cultivated a business dispatch which will receive general commendation. The whole of the task committed to the Committee is by no means accomplished, but having become convinced by the evidence already submitted to them that on a point of great importance a radical change is needed, they have promptly said so, and this in terms which cannot be mistaken. When, further, we learn that the War Office agrees with the Committee it may be assumed that the case is a very strong one, more particularly as the new proposal is to take from the War Office functions which have hitherto been exercised under military discipline and control. The Committee thus at the outset appears to score a large triumph and to justify both its existence and the methods it has pursued. The Secretary for War welcomes its proposals "with both hands," and the Prime Minister and the Director-General of Recruiting join in the chorus of approval.

The substance of the Committee's recommendation is that the whole organisation of recruiting medical boards and of the medical examinations and re-examinations shall be transferred from the War Office to the Local Government Board. The proposal is indeed a tremendous one. It means that from the investigations preliminary to the entry of men into the Army the military element entirely disappears. Only when a civilian judgment has decided on his fitness will a man come within the grasp of Army discipline. The members of the examining authority will not be clothed in uniform nor will a military president appear at their deliberations. Whether the decision is for admission or rejection it will be a civilian decision—a decision in which the Army has no voice and over which the Army has no powers of review. It is almost staggering to read that the War Office assents to such a proposal, and even assents to it gladly. Nay, more: the Secretary for War wishes to go further and to hand over to a civilian authority not merely the medical judgment of conscripted men but the whole business of recruiting—the registration of the available men, the summoning of them for examination, the organisation of the medical lists, the co-ordination of these in relation to the requirements of the Army, and a review of the circumstances of each man in relation to the importance and urgency of his civil calling.

At first sight, it must be admitted, there is an element of surprise in the announcement that the War Office is willing and even anxious to release from its authority an area over which up to the present it has exercised almost absolute sway. Yet the desire may fairly be described as having an eminently common-sense quality. In happier days every recruit was essentially a volunteer, and the Army authorities only excited his ire when they refused to accept him. But now complaint is aroused, when it is aroused at all, by a decision in the opposite direction. It is the man who is taken when he thinks he ought to be refused who feels the bitterness of the position. A similar attitude of mind exists in men snatched by the military machine from homes and occupations in which it is their personal interest to remain. Under the existing arrangements the odium of all these proceedings necessarily falls upon the Army, and in recent months, in consequence of the re-examinations legalised by the Review of Exceptions Act, the numbers of the discontented and the acuteness of their complaints have been much increased. Whether the grievances of these several classes are or are not well-founded does not affect the position that the Army is regarded as responsible for them, and in the numerous circles in which these grievances circulate it is on the Army that the blame is placed. Obviously, if the War Office can transfer all this hostile criticism to other shoulders it is but natural it should do so, and hence it is easy to believe that in this respect the military will be glad to see some civilian authority set in the forefront of the battle. For it may be taken as certain that whatever organisation is charged with the selection of recruits there must in the existing circumstances be a considerable volume of dissatisfaction. Men must be obtained unless the national cause is to perish. They cannot be obtained without much inconvenience and loss and unhappiness to many individuals. The choice lies between two evils, and one or the other must be accepted. The circumstances of the hour place personal interests in the background, but the sacrifice for the individuals concerned is in many instances not less a bitter one, and the organisation which is the immediate agent of compulsion must suffer a wide unpopularity. Like less dignified organisations the War Office sensibly prefers that the unpopularity shall be upon "the other man."

In saying this, however, there is no suggestion that so narrow a view is the sole motive of the authorities. These, equally with the Parliamentary Committee, may be presumed to be actuated by considerations of the public interest. The evidence submitted to this Committee has shown plainly that as the civilian population has to be more and more drastically searched in order to find the men required there is a corresponding extension of objection to the process. In all the proceedings it is the Army which is in the public gaze, and it is a fair question whether such a position is a wise and defensible policy. Inevitably, as the Army demands the men and uses its own agents to secure them there grows up a belief that the methods employed are not characterised by undue scruples. Such, broadly, is the position to which we have come, and there are large and sound reasons why an effort should be made to modify it. This, indeed, is the proclaimed motive of the Parliamentary Committee in advising that a change be made, and that it be made without delay. It is " in order to restore public confidence " that they propose the prompt transference of the keeping of the gateways which lead to the Army from the present military guardians to the care of a civilian authority. To read into this proposal a censure on the War Office authorities is, however, an unjustifiable conclusion. No doubt the evidence given before the Parliamentary Committee has shown that pressure has been exerted from the centre on the medical examining boards, and the official doctrine that every man who can work for his living as a civilian can work with equal success in the Army has excited a degree of public distrust. But so long as the War Office has not only the responsibility of using the men but also the duty of finding them it is inevitable that it should apply the methods which seem to be necessary to this end. The pressure exerted is not a mere exercise of arbitrary power for its own sake. It is events rather than individuals which impose it. Hence it will continue, whatever be the authority which is charged with the provision of recruits in numbers sufficient to meet the national emergency. We always come back to the original dilemma. Either men must be found or our armies cannot continue to hold the field. Between these alternatives the choice has to be made, and as it cannot be doubted that the nation is determined to continue the struggle until victory crowns our arms, the simple conclusion is that soldiers must be found for the ranks. What this means to many we know only too well. But the necessity is upon us either to conquer or to submit. The nation is resolved to conquer. Therefore it must provide the soldiers. What is now suggested will not in the least affect this necessity. But it will alter the method by which the necessity is to be met.

Apart from the convenience of the War Office, and apart also from any question of individual blunders and errors and stupidities, there is much to be said for the new proposal on the broadest possible grounds. The first step in national policy is taken by civilian authority, which determines, as the case may be, the decision of peace or war. It is for the Army experts in the event of the latter to say what numbers of men will be needed to translate the civilian decision into action. Upon such a point their decision is supreme. But the provision of the men is not a natural function of such experts. Clearly this lies with those who have decreed the policy and decided the aim. From various circumstances this division of labour has not been strictly followed, and the present agitation is largely an outcome of this mistaken and unnatural method. The Army has been pushed into a position of prominence relative to the obtaining of recruits which it never ought to have occupied, and it has thus come to hold in the view of certain sections of the community a status comparable to that held in former days by the press-gang. This is both unfair to the Army and prejudicial to the public interest, and its rectification may well therefore attract the good will of all loyal citizens. The proposal of the Parliamentary Committee by removing all military influence from the medical examinations will do something to restore the natural and proper relationship of the Army to the nation, but the wider suggestion of the Secretary for War is needed for the full accomplishment of this end. Whether this suggestion is or is not to be adopted remains to be seen.

Whoever else may be satisfied or dissatisfied with the new arrangements, the doctors doubtless will welcome them; and this both on the military and the civilian side. In the Director-General's department there may well be satisfaction at relief from a responsibility which must have been singularly oppressive. What with demands from above and complaints from below the lot of the heads of the R.A.M.C. must for a long time have been anything but a happy one. Of their failures and mistakes all the world is informed, but of their achievements in the provision of recruits little is said. This latter duty they will now doubtless gladly transfer to other hands. Not less satisfied, we imagine, will be the doctors who have been obliged to don a temporary uniform and who may henceforth resume their civilian status. No man can be quite content to be compelled to appear in the garb of a profession which is not his own and to take on a pomp and circumstance which are foreign to his training. In its place this decorative aspect has its value, and even its necessity. But civilian practitioners suddenly thrust into it cannot but find it irksome. There is an element of the unreal about such a position, and the newcomer, supreme in his own field, is naturally not quite welcome to those who have passed through all the subordinate grades. Above all, the civilian doctor decorated for purposes of recruiting will now resume the freedom and independence to which he is accustomed. He will accept a full measure of personal responsibility, and will appear as having the dignity of his own profession instead of the status of an amateur soldier. These are not times for petty squabbles, but human nature has to be reckoned with even in times of national emergency, and some of the disputes related in the press are almost in the nature of things. Still more important is the practical certainty that under the new conditions the work may be expected to be performed more satisfactorily. Medicine does not run well in official fetters, and men accustomed to its large liberty do not easily adjust themselves to rigid rules and formal regulations. Professional work conducted under pressure is rarely the best work, and therefore on the score of efficiency as well as in the interests of the Army and of the nation generally it is all to the good that the medical profession shall be free to conduct the examination of recruits under conditions in harmony with its own traditions and methods. The success of these in other directions will now be applied to the medical aspect of recruiting.

